# Risk factors associated with exposure to bovine respiratory disease pathogens during the peri-weaning period in dairy bull calves

**DOI:** 10.1186/s12917-018-1372-9

**Published:** 2018-02-27

**Authors:** Gerard M. Murray, Simon J. More, Tracy A. Clegg, Bernadette Earley, Rónan G. O’Neill, Dayle Johnston, John Gilmore, Mikhail Nosov, Máire C. McElroy, Thomas J. Inzana, Joseph P. Cassidy

**Affiliations:** 1 Sligo Regional Veterinary Laboratory, Department of Agriculture, Food and Marine, Doonally, Sligo, Ireland; 2 Centre for Veterinary Epidemiology and Risk Analysis, UCD School of Veterinary Medicine, University College Dublin, Belfield, Dublin 4 Ireland; 3 Animal and Bioscience Research Department, Animal & Grassland Research and Innovation Centre, Teagasc, Grange, Dunsany, Co. Meath, Ireland; 4 Central Veterinary Research Laboratory, Department of Agriculture, Food and Marine, Backweston, Celbridge, Co. Kildare Ireland; 5 Farmlab Diagnostics, Emlagh Lodge, Elphin, Co. Roscommon Ireland; 6 Department of Biomedical Sciences & Pathobiology, Virginia-Maryland College of Veterinary Medicine, Virginia Tech, Blacksburg, VA USA; 7 School of Veterinary Medicine, University College Dublin, Belfield, Dublin 4 Ireland

**Keywords:** Calves, Antibodies, Bovine respiratory disease, Dairy, Exposure

## Abstract

**Background:**

Bovine respiratory disease (BRD) remains among the leading causes of death of cattle internationally. The objective of this study was to identify risk factors associated with exposure to BRD pathogens during the peri-weaning period (day (d)-14 to d 14 relative to weaning at 0) in dairy bull calves using serological responses to these pathogens as surrogate markers of exposure.

Clinically normal Holstein-Friesian and Jersey breed bull calves (*n* = 72) were group housed in 4 pens using a factorial design with calves of different breeds and planes of nutrition in each pen. Intrinsic, management and clinical data were collected during the pre-weaning (d − 56 to d − 14) period. Calves were gradually weaned over 14 days (d − 14 to d 0). Serological analysis for antibodies against key BRD pathogens (BRSV, BPI3V, BHV-1, BHV-4, BCoV, BVDV and *H. somni)* was undertaken at d − 14 and d 14. Linear regression models (for BVDV, BPI3V, BHV-1, BHV-4, BCoV and *H. somni)* and a single mixed effect random variable model (for BRSV) were used to identify risk factors for changes in antibody levels to these pathogens.

**Results:**

BRSV was the only pathogen which demonstrated clustering by pen. Jersey calves experienced significantly lower changes in BVDV S/P than Holstein-Friesian calves. Animals with a high maximum respiratory score (≥8) recorded significant increases in *H. somni* S/P during the peri-weaning period when compared to those with respiratory scores of ≤3.

Haptoglobin levels of between 1.32 and 1.60 mg/ml at d − 14 were significantly associated with decreases in BHV-1 S/N during the peri-weaning period. Higher BVDV S/P ratios at d − 14 were significantly correlated with increased changes in serological responses to BHV-4 over the peri-weaning period.

**Conclusions:**

Haptoglobin may have potential as a predictor of exposure to BHV-1. BRSV would appear to play a more significant role at the ‘group’ rather than ‘individual animal’ level. The significant associations between the pre-weaning levels of antibodies to certain BRD pathogens and changes in the levels of antibodies to the various pathogens during the peri-weaning period may reflect a cohort of possibly genetically linked ‘better responders’ among the study population.

## Background

Bovine respiratory disease (BRD) remains among the leading causes of death of dairy and beef cattle of all ages in Ireland [1] and internationally [2]. Dairy calf pneumonia (enzootic calf pneumonia) represents an epidemiologically distinct component of the BRD complex, typically affecting 2 to 6 month old calves; shipping fever of feedlot cattle and atypical interstitial pneumonia are other recognised syndromes [3].

While indoor or outdoor individual housing of calves has been recognised as beneficial to dairy calf health [4] legislative changes in animal welfare in Ireland and Europe (EU Directive 91/629/EC and EU Decision 97/182/EC) have encouraged group housing of calves, which also facilitates more efficient use of labour and space. However, this shift has presented the dairy industry with new challenges in disease control [5] and increased the risk of BRD among young dairy calves [6–8].

Stress is an important co-factor in the pathogenesis of BRD [9, 10]. Clinically affected calves shedding large numbers of pathogens into the environment act as an important source of exposure for other calves. In addition, BRD pathogens can also be carried and shed by apparently healthy animals [11, 12]. Weaning has been traditionally recognised as a stressful time for calves with both nutritional and non-nutritional factors contributing to weaning distress [13]. Non-nutritional factors such as the breaking of the maternal bond and the rearrangement of the social group tend to be critical factors for beef suckled calves in particular. As dairy calves are generally separated from the dam shortly after birth, weaning distress in the dairy calf typically arises predominantly from nutritional factors [14]. When coupled with the increased interaction and contact of calves in group housing, weaning distress can facilitate the efficient spread of BRD pathogens leading to exposure and, potentially, disease. Inevitably there are interactions and cross-effects between pathogens which together lead to variable and complex immunological and pathological effects in each calf.

Many studies have examined the effects of intrinsic [15], management [16, 17] or clinical [18] risk factors for BRD; however, reports as to the various potential risks these variables pose in exposing calves to BRD pathogens are lacking. The objective of this study was to identify risk factors associated with exposure to BRD pathogens during the peri-weaning period in dairy bull calves using serological responses to these pathogens as surrogate markers of exposure. These responses were determined by calculating changes in sample to positive ratios (S/P), percentage positivity (PP) or sample to negative ratios (S/N), to a wide range of recognised BRD pathogens during the peri-weaning period (day (d) -14 to d 14 relative to weaning at d 0).

## Methods

### Animal management

This study was conducted as part of a larger study designed to examine changes in haematological profiles and gene expression in response to gradual weaning. Ethical approval for this study was sought, and received, from the Teagasc Animal Ethics Committee. Animal management, sample collection and haematological analysis have been outlined previously by Johnston et al. [14]. Briefly, 72 clinically normal bull calves of Jersey or Holstein-Friesian breed were sourced from 2 preferential supplier farms at a mean age of 19 (S.D. 8) days and were group housed indoors in 4 sawdust-floored pens (16, 18, 18 and 20 per pen) from d − 56 (relative to weaning (d 0)) to d 28 of the study. A sample size calculation for linear regression based on a power level of 0.8, significance level of 0.05 and a range of anticipated effects and numbers of predictors suggested a minimum sample range of 59 to 65 animals. Calves were immunised on arrival against bovine herpesvirus 1 (BHV-1; Rispoval IBR Marker Live administered intramuscularly, Zoetis), and, using a combined vaccine (Bovilis Bovipast RSP, MSD, inactivated vaccine administered subcutaneously), they were also immunised against bovine parainfluenza 3 virus (BPI3V), bovine respiratory syncytial virus (BRSV), and *Mannheimia haemolytica* serotypes A1 and A6. A single combined booster dose against BRSV, BPI3V and *Mannheimia haemolytica* was administered 4 weeks later. Vaccination against *Salmonella* Dublin and *Salmonella* Typhimurium (Bovivac S, inactivated vaccine administered subcutaneously) was also administered on arrival. The vaccination status of the dams was not available. The study was structured as a factorial design with two breeds (Holstein-Friesian and Jersey), and three planes of nutrition (high (H), medium (M) and low (L)) within each breed and calves were stratified to a nutrition treatment within each breed, on the basis of live-weight, age at the first day of the study (d − 56) and sire [14]. Each pen contained calves of each breed and each plane of nutrition and calves were fed using automatic milk (Vario Powder; Förster-Technik GmbH, Engen, Germany) and concentrate (KFA3-MA3; Förster-Technik GmbH) feeders. All calves were offered approximately 400 g straw daily, from a rack within the group pen during the peri-weaning period. During the pre-weaning period (d-56 to d-14), Holstein-Friesian calves on the H, M and L planes of nutrition were offered 1.2 kg milk replacer (8 l at 150 g/l) with ad libitum concentrate, 0.8 kg milk replacer (6 l at 133.33 g/l) with a maximum of 1.5 kg concentrate and 0.5 kg milk replacer (4 l at 125 g/l) with a maximum of 1 kg concentrate, daily, respectively. The Jersey calves on the H, M and L planes of nutrition were offered 0.8 kg milk replacer (6 l at 133.33 g/l) with ad libitum concentrate, 0.5 kg milk replacer (4 l at 125 g/l) with a maximum of 1.5 kg concentrate and 0.35 kg milk replacer (3.5 l at 100 g/l) with a maximum of 1 kg concentrate, daily, respectively. During the weaning phase (d-14 to d0), daily milk replacer was gradually reduced and by d − 1, all calves had been consuming at least 1 kg of concentrate per day for 3 consecutive days. On d 0, milk replacer was eliminated from the diet of all calves.

Animals were maintained on different planes of nutrition which were devised for each breed using National Research Council guidelines [19] to achieve a target growth rate of ⩾1.0, 0.7 and < 0.5 kg/day, for Holstein-Friesian breed calves on the H, M and L planes of nutrition and a target growth rate of 0.7, 0.5 and ⩽0.3 kg/day, for Jersey breed calves on the H, M and L planes of nutrition, respectively [14].

### Clinical assessment

Clinical assessments were carried out on all calves twice weekly during the pre-weaning and weaning periods (d − 56 to d 0). A modified version of the Wisconsin health scoring criteria was used to score clinical criteria [20]. A cumulative respiratory score (0–12) was devised from nasal discharge (0–3), eye (0–3) or ear (0–3) score (whichever was greatest), cough index (0–3) and rectal temperature based on the method described by Lago et al. [21]; the maximum respiratory score achieved by each calf between d − 56 and d − 14 was recorded.

### Sampling

Blood samples were collected from all calves on arrival, and on d − 14 and d 14 relative to weaning (d 0), via jugular venipuncture. Serum was harvested and samples were stored at − 20 °C pending analysis. Blood samples were also collected in 6 ml K_3_ Ethylenediaminetetraacetic acid (K_3_EDTA) tubes (Vacuette; Cruinn Diagnostics, Dublin, Ireland) and in 9 ml Lithium Heparin (LH) tubes (Vacuette; Cruinn Diagnostics) on d − 14 and d 14 relative to weaning (d 0) for haematological analysis and to determine the concentration of the acute phase protein, haptoglobin respectively. LH blood samples were centrifuged at 4 °C (1600×g for 15 min) and the plasma was harvested and stored at − 20 °C until analysed.

### Serological testing

The K_3_EDTA blood samples were analysed immediately after collection using an ADVIA 2120 analyser (AV ADVIA 2120; Bayer Healthcare, Siemens, UK), containing software necessary for the analysis of bovine blood as described by Johnston et al. [14]. Zinc sulphate turbidity (ZST) test analysis was carried out at 520 nm using a spectrophotometer [22]. The haptoglobin concentration was measured using an automatic analyser (Olympus AU 400 Analyser; Beckman Coulter, Inc., Clare, Ireland) and a commercial assay kit (Tridelta Development Ltd., Maynooth, Ireland).

Serum samples were analysed for the presence of immunoglobulin (Ig) G against BRSV, BPI3V, BCoV (SVANOVA Biotech, Uppsala, Sweden) BHV-4 (Bio – X Diagnostics S.A., Rochefort, Belgium), BVDV (IDEXX Laboratories Inc., Maine, USA) and BHV-1 glycoprotein E (IDEXX Laboratories Inc., Maine, USA) by commercially available enzyme-linked immunosorbent assays (ELISA) each of which were performed in accordance with the manufacturer’s instructions. *H. somni* serology was performed by an in-house ELISA to detect IgM/IgG against *H. somni* exopolysaccharide (EPS) as described by Pan et al. [23]. Details on the calculations used in the ELISA analyses, cut-off values employed and the correlation between ELISA results and changes in antibody status are presented in Table 1.

**Table 1:** Details on the calculations used in the ELISA analyses, the cut-off values employed and the correlation between the ELISA results and antibody levels

ELISA	Optical density (OD) measured at	Calculation used	Positive results	Correlation between ELISA value and antibody level
BRSV	450 nm	PP = Sample_COD_ /P_COD_ X100	PP ≥ 10	Positive
BPI3V	450 nm		PP ≥ 10	Positive
BCoV	450 nm		PP ≥ 10	Positive
BHV-1 gE	650 nm	S/N ratio = Sample_OD_/N_2_	S/*N* ≤ 0.6	Negative
BHV-4	450 nm	S/P ratio = (Sample_OD_ – N_1_)/(P_1_-N_1_)	S/*P* ≥ 0.3	Positive
BVD	450 nm	S/P ratio = (Sample_OD_ – N_2_)/(P_2_-N_2_)	S/P ≥ 0.3	Positive
*H. somni*	450 nm	S/P ratio = (Sample_OD_ – N_3_)/(P_3_-N_3_)	S/*P* > 0.6	Positive

Note that the subsequent computation applied to the raw Optical Density (OD) result can differ between ELISA kits and has been designed to give optimal diagnostic performance. Therefore the different comparison parameters (e.g. S/N, S/P or PP values) used here are inherent to the test rather than to the pathogen

COD = corrected optical density (calculated by subtraction of the mean OD value for the negative control)

Positive control (P_1_), Mean of 2 (P_2_) or 3 (P_3_) positive controls

Negative control (N_1_), Mean of 2 (N_2_) or 3 (N_3_) negative controls

### Statistical methods

Separate multivariable linear regression models (for BHV-1, BHV-4, BoCV, BPI3V, BVDV, BRSV and *H. somni*) were developed to quantify the effects of the various risk factors on the exposure of the calves to BRD pathogens between d − 14 and d 14. A plot of the residuals of a simple model for each dependent variable was created against the predicted values of that model and the normality of the residuals was assessed to determine if a transformation of the dependent variable was warranted; the optimal transformation being determined by Tukey’s ladder of powers [24]. As the diseases studied are transmissible, the potential for clustering within pens (i.e. group) was accounted for by including pen as a random effect for each of the outcome variables examined and its significance was tested using a likelihood ratio test. For outcome variables in which clustering by pen was not significant (*p* < 0.05), a linear regression model was preferred. The change in antibody status, as reflected by changes in PP values (BPI3V, BCoV and BRSV), S/P ratios (BHV-4, BVDV and *H. somni*) or S/N (BHV-1) ratios on ELISA analyses, between d − 14 and d 14 was the dependent variable in each analysis. The S/P ratios, S/N ratios or PP values at d − 14 of each BRD pathogen was considered as a risk factor for changes in the antibody levels against other BRD pathogens in the analysis during the peri-weaning period (d − 14 to d 14) but were not included in the analysis for that specific pathogen (i.e. BHV-1 S/N at d-14 was not considered as a risk factor for BHV-1 S/N change during the peri-weaning period). Univariable analyses were performed individually to determine the association of each potential risk factor with the dependent variable using Stata v11. The risk factors included in the univariable analysis are listed in Table 2.

**Table 2:** The potential risk factors for exposure to BRD pathogens (as reflected by S/P ratio (for BHV-4, BVDV, *H. somni*), PP value (for BRSV, BPI3V, BCoV) and S/N ratio (BHV-1)) which were included in the univariable analysis

Risk factor type	Details
Intrinsic risk factors	Calf breed (Jersey or Friesian)
	ZST score on arrival
	Arrival age
	Arrival weight
Management risk factors	Pen to which the animal was assigned (*n* = 4)
	Nutrition plane (high, medium or low)
	UFL (*unité* fourragère *lait)* intake in the pre-weaning period (d-56 to d-14)
	Percentage of UFL allowance consumed in pre-weaning period (d-56 to d-14)
	Average daily gain in pre-weaning period (d-56 to d-14)
Clinical risk factors	Maximum temperature recorded during the pre-weaning period (d-56 to d-14)
	Mean temperature recorded during the pre-weaning period (d-56 to d-14)
	Maximum respiratory score in the pre-weaning period (d-56 to d-14)
	Number of times treated for BRD in pre-weaning period (d-56 to d-14)
	Number of sickness bouts recorded in the pre-weaning period (d-56 to d-14)
	Total white blood cell count at d − 14
	Lymphocyte count at d − 14
	Haptoglobin level at d − 14
	*H. somni* S/P at d − 14
	BHV-4 S/P at d − 14
	BVDV S/P at d − 14
	BHV-1 S/N at d − 14
	BPI3V PP at d − 14
	BRSV PP at d − 14
	BCoV PP at d − 14

The pre-weaning period was from d − 56 to d − 14 relative to weaning at d 0. Note that BRD pathogen S/P ratios, S/N ratios or PP values at d − 14 were not considered as risk factors for changes in antibody levels for that specific pathogen during the peri-weaning period (d − 14 to d 14)

Risk factors identified as significant in the univariable analyses (*p* < 0.20) were included in the multivariable analysis. Independent variables were plotted against the dependent variable and, if a linear relationship did not exist between both and a transformation of the independent variable was not appropriate due to a non-linear relationship between the transformed independent variable and the dependent variable, continuous independent variables were then included as categorical variables based on the quartiles of the continuous independent variable. Plausible interactions were considered in the full model during model construction and two-way interactions identified as significant (i.e. between arrival age, arrival weight, ZST and breed) were included in the multivariable model during development. The variance inflation factor was used to detect multicollinearity. A backward stepwise selection procedure based on a likelihood ratio test (*p* > 0.05) was used to eliminate terms from the model. Regression diagnostics (heteroskedasticity, skewness and kurtosis) and plots of the residuals versus the predicted values were used to assess the presence of outliers and to check the fit of the final model.

## Results

**Fig. 1:**
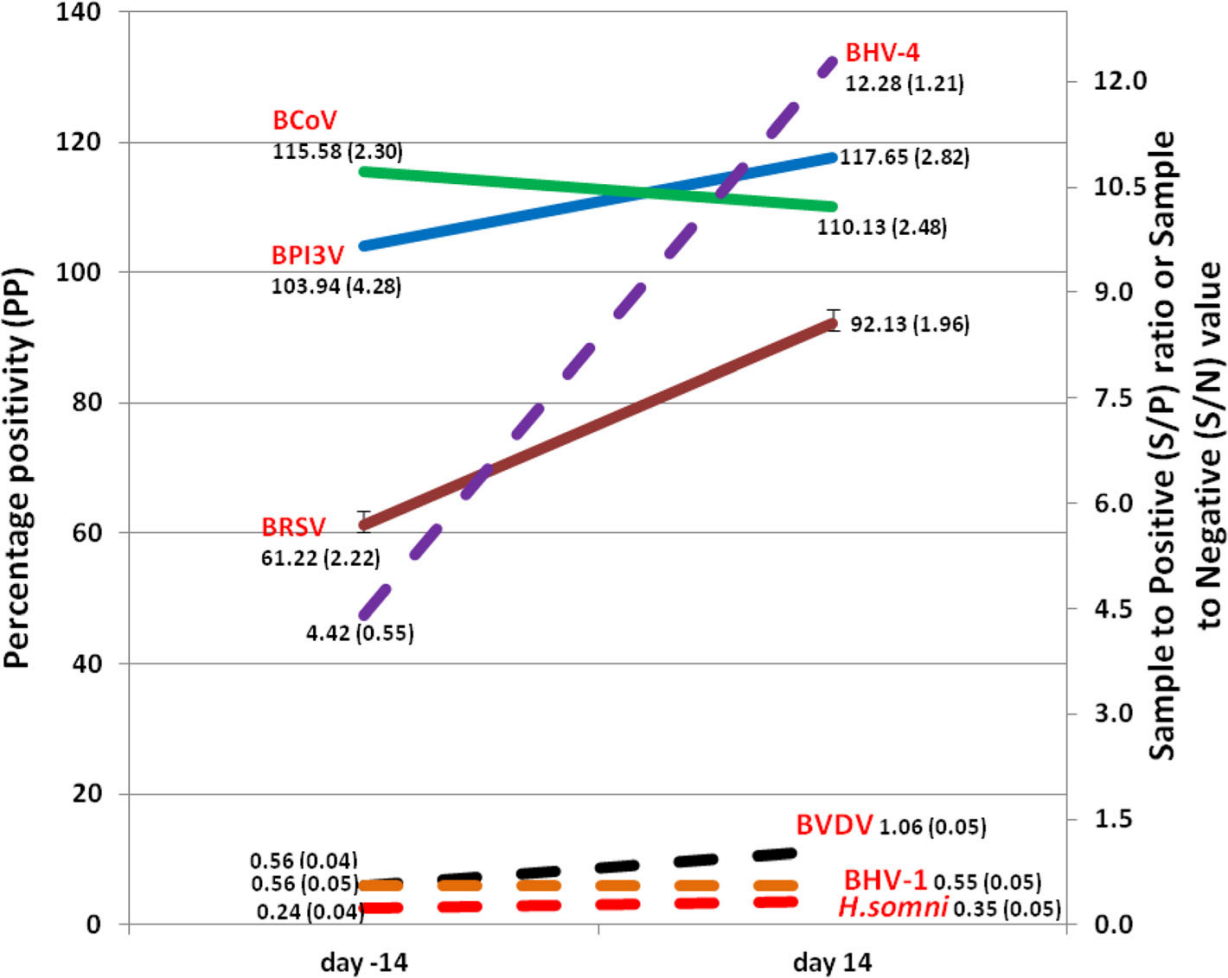
The mean values of the antibody levels (as reflected by S/P. PP or S/N values) to each of the BRD pathogens of interest and the standard error of the mean (in parantheses) at d − 14 and d 14 relative to weaning at d 0. Note that solid lines (BCoV PP, BPI3V PP, BRSV PP) are plotted against the left hand y-axis while the broken lines (BVDV S/P, BHV-4 S/P, BHV-1 S/N and *H. somni* S/P) are plotted against the right hand y-axis

Of the 72 calves in the study, 43 were Holstein-Friesian and 29 were Jersey calves. In total, 24 animals were on the high plane of nutrition with 25 and 23 on the medium and low planes of nutrition respectively. The mean age and weight of calves on arrival were 19 (S.D. 8) days and 41.4 (S.D. 8.4) kgs respectively and the mean ZST result on arrival was 17.95 units (SD 3.46 units). A bout of sickness was defined as a concurrent recorded temperature > 39.5 °C and respiratory score of ≥5 in a calf. Sickness was not recorded during the pre-weaning period (d − 56 to d − 14) in 36 calves, was recorded once in 26 calves, twice in 7 calves, 3 times in 2 calves and 4 times in 1 calf. The mean values of S/P, PP or S/N for each of the respective BRD pathogens of interest and the change in mean values from d − 14 to d 14 (relative to weaning at day 0) are presented in Fig. 1.

**Fig. 2:**
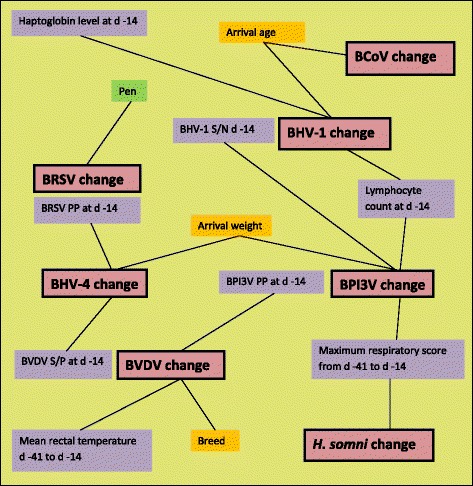
A schematic diagram of the significant linkages identified in the seven models between intrinsic (orange boxes), management (green box) and clinical (purple boxes) independent variables and the dependent (pink boxes) variables. The dependent variables were antibody level changes during the peri-weaning period (d − 14 to d14 relative to weaning at d 0) as reflected by changes in S/N ratio (for BHV-1), PP value (for BRSV, BCoV and BPI3V) or S/P ratio (for BHV-4, BVDV and *H. somni*)

A schematic diagram of significant linkages identified in the seven regression models is presented in Fig. 2. The model for the change in BRSV PP during the peri-weaning period included the pen the animal occupied as a random effect. Transformed outcome variables were used in the models of BVDV S/P change (square of the outcome variable), BHV-1 S/N change (cubed root of the outcome variable) and *H. somni* S/P change (cubed root of the outcome variable). The variables which were present in each model are displayed in Table 3.

**Table 3:**
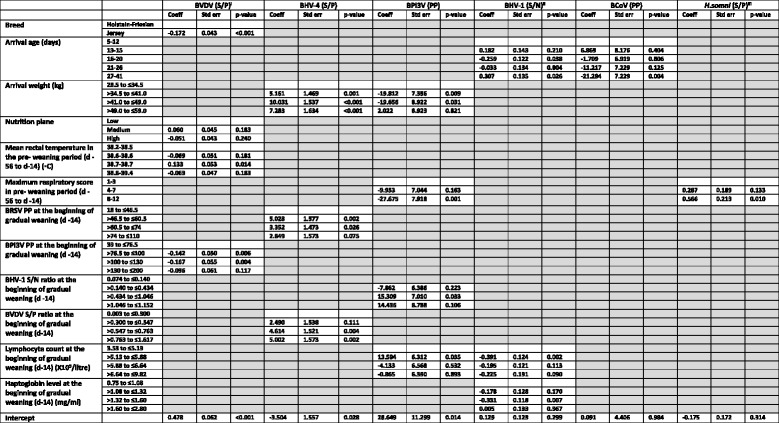
Variables significantly associated in a multivariable linear regression model with a change in respiratory pathogen serology percentage positivity (PP), sample to positive ratio (S/P) or sample to negative control ratio (S/N) from the beginning of gradual weaning (day − 14) to day 14 post weaning (day+ 14) in housed dairy calves

Note that boxes coloured in grey denote variables which were not significant (*p* > 0.05) in the multivariable model of a given pathogen

^a^The square of BVDV percentage positive change was used as the dependent variable

^b^The cubed root of the change in sample to negative control ratio was used as the dependent variable

^c^The cubed root of the change in sample to positive control percentage was used as the dependent variable

Breed (*p* < 0.001) and higher BPI3V P *P* values at d − 14 (*p* ≤ 0.006) were significantly associated with changes in the square of BVDV S/P ratios during the peri-weaning period (d − 14 to d 14 relative to weaning at d 0). Jersey breed calves experienced a significantly reduced change in squared BVDV S/P value compared to Holstein-Friesian calves as did calves with higher BPI3V PP values (between 76.5 and 130) at d − 14 compared to those with lower BPI3V PP values (between 39 and 76.5). A mean rectal temperature of 38.7 °C during the pre-weaning period was also significantly associated (*p* = 0.014) with changes in the square of BVDV S/P (Table 3).

Arrival weight was a significant risk factor (*p* ≤ 0.001) for BHV-4 S/P change during the same period with calves between 34.5 kg and 59 kg all experiencing greater increases in BHV-4 S/P when compared to lighter calves (between 28.5 and 34.5 kg). BRSV PP values between 46.5 and 74 on d − 14 were also significantly associated (*p* ≤ 0.026) with increases in BHV-4 S/P ratios during the peri-weaning period when compared to those with BRSV PP values between 18 and 46.5. Likewise, higher BVDV S/P ratios (between 0.547 and 1.617) at d − 14 were significantly associated (*p* ≤ 0.004) with increases in BHV-4 S/P ratios during the peri-weaning period when compared to calves with lower BHV-4 S/P ratios (≤0.300).

A smaller BPI3V PP change during the peri-weaning period was significantly associated (*p* ≤ 0.031) with a heavier arrival weight (34.5–49 kgs) among calves entering the calf house when compared to lighter calves. The reduced change in BPI3V PP value was also associated (*p* = 0.001) with a high respiratory score (≥8) during the pre-weaning period (d-56 to d-14) when compared to calves with the lowest respiratory scores (≤3). Increases in BPI3V PP values were associated (*p* = 0.033) with BHV-1 S/N ratios at d − 14 of > 0.434 and ≤1.046 when compared to lower S/N ratios between 0.074 and 0.140. Lymphocyte counts at the beginning of weaning (d − 14) of > 5.13 X10^9^/l to ≤5.88 X10^9^/l were also significantly associated with an increase in the change of BPI3V PP value (*p* = 0.035) during the peri-weaning period, when compared to those with a lymphocyte count of ≤5.13 X10^9^/l.

BHV-1 S/N ratios decreased (*p* = 0.038) during the peri-weaning period in calves that arrived at 16–20 days of age when compared to those aged 5–12 days on arrival. This change was reversed in calves aged 27 to 41 days of age on arrival (*p* = 0.026) with an increase in BHV-1 S/N ratios when compared to the youngest arriving calves (aged 5–12 days). Decreases in BHV-1 S/N ratios during the peri-weaning period were significantly associated with haptoglobin levels of between 1.32 and 1.60 mg/ml (*p* = 0.007) when compared to haptoglobin levels between 0.75 and 1.08 mg/ml at the beginning of weaning (d − 14). Lymphocyte counts at the beginning of weaning (d − 14) between 5.13 X10^9^/l and 5.88 X10^9^/l were significantly associated with a decrease in BHV-1 S/N value (*p* = 0.002) during the peri-weaning period when compared to those with lower lymphocyte counts (≤ 5.13 X10^9^/l).

Changes in the BCoV P*P* values during the peri-weaning period were significantly less (*p* = 0.004) in the oldest calves (aged 27–41 days) when compared to the youngest (aged 5–12 days) calves on arrival.

A maximum respiratory score of ≥8 prior to the commencement of gradual weaning was significantly associated (*p* = 0.010) with an increase in the change of *H. somni* S/P value in the peri-weaning period. Changes in BRSV PP value during the same period were associated with the maximum rectal temperature recorded during the pre-weaning period and were also significantly clustered (*p* < 0.001) by the pen the calves occupied prior to the commencement of gradual weaning (Table 4).

**Table 4:** Linear mixed regression model of the variables associated with a change in BRSV percentage positivity (PP) from the beginning of gradual weaning (day -14) to 2 weeks post weaning (day +14)

Variable	Categories	Coefficient	Standard error	*P* value
Intercept		31.577	5.292	<0.001
Maximum rectal temperature (°C) in the pre-weaning period (d-56 to d-14)	38.9–39.4	Baseline		
	39.5–39.6	7.956	4.955	0.108
	39.7–40.0	-7.442	4.513	0.099
	40.1–41.3	-3.732	4.474	0.404
Random effects		Variance estimate	Standard error	
Pen		78.259	73.088	<0.001
Residual		194.018	34.026	

## Discussion

The objective of this study was to identify risk factors associated with exposure to BRD pathogens during the peri-weaning period in dairy bull calves using serological responses to these pathogens as proxy indices of exposure. These responses were determined by calculating changes in sample to positive ratios (S/P), percentage positivity (PP) or sample to negative ratios (S/N), to a wide range of recognised BRD pathogens during the peri-weaning period. The calves in this study were housed in a situation of minimum stress and management advantage to reduce adverse effects from those sources on our findings. By focusing less on disease but rather on good health, and factors influencing the calf immune response, our study identified intrinsic (breed, arrival age and arrival weight), management (pen) and clinical (rectal temperature and respiratory score) factors which were significantly associated with changes in BRD pathogen serological status.

Arrival age at housing was significantly associated with a reduction in BCoV PP (*p* = 0.004) and an increase in BHV-1 S/N (*p* = 0.026) in the oldest cohort (27–41 days) when compared to the youngest cohort (5–12 days) of calves. As both of these results are consistent with a decrease in antibody levels, it is likely that they reflect the natural decline of maternally derived antibody (MDA) with age. BCoV has been identified as a prevalent pathogen in BRD in Ireland [25] and internationally [26, 27]. Recently, Toftaker et al. [28] in a bulk tank serology cross-sectional study in Norway, identified herd size, purchase of livestock, geographic location, proximity to neighbours and seropositivity to BRSV as risk factors for seropositivity to BCoV in dairy herds. As acknowledged by Pardon et al. [29], BCoV seroconversion does not distinguish between respiratory or enteric infections, although concurrent faecal and nasal shedding is a relatively frequent event in calves [30]. Seventeen calves in the study population were recorded with a faecal score of ‘2’ (loose and watery faeces) suggestive of enteric infection during the pre-weaning period. As BCoV infection typically causes diarrhoea in calves between 3 and 21 days of age [31], it is quite likely that younger calves in this study housed at the peak age of enteric infection were more likely to acquire and spread BCoV infection than older animals arriving after the peak of enteric infection had passed.

In this study we recorded lower unit increases in BPI-3 V PP and higher unit increases in BHV-4 S/P during the peri-weaning period among heavier calves on arrival into the house when compared to the lightest group. Previously, low bodyweight at housing has been identified as a risk factor for the development of BRD at the individual [32, 33] but not at the group [34] level. While the reported BHV-4 S/P increase would appear to contradict these findings, it is important to recall that BRD pathogen antibodies reflect exposure history rather than disease. One possibility is that lower weight calves were exposed and seroconverted to BHV-4 at an earlier stage than heavier calves and the change in antibody level during the peri-weaning period reflects delayed exposure in heavier calves. This may be, in part, due to more prolonged systemic effects of BHV-4 infection as a result of its capability for latency or inherent host animal traits such as the respiratory rate or the metabolism of heavier calves may also play a role. Alternatively, lower bodyweight calves may not be capable of mounting an equivalent antibody response to that of heavier animals [35]. Further focused research examining the mechanism of the effect of bodyweight on calf immune response to BRD pathogens is warranted.

The finding of a clustering effect of exposure to BRD pathogens would seem intuitive. Brscic et al. [34] reported a higher prevalence of respiratory distress in young calves when the number of animals per pen increased. Miller et al. [36] reported that BRD outbreaks tend to cluster within calf housing systems; however, of the BRD pathogens examined in the present study, only changes in BRSV PP were significantly clustered by pen. This may indicate that BRSV is significant at the group rather than the individual level. MDA can suppress responses to BRSV resulting in a minority of BRSV seronegative calves seroconverting. Such an event may have impacted the detection of associations between risk factors and changes in BRSV PP in the current study [29, 37–39].

During the pre-weaning period, a high maximum respiratory score (≥8), reflecting typical clinical signs of BRD, was significantly associated (*p* = 0.001) with a decrease in BPI3V PP during the peri-weaning period. While this result may appear counter-intuitive, a similar finding was reported by Tuncer and Yesilbag [40]. In that study, despite an increase in the proportion of BPI3V seropositive calves between sampling points at 1 and 2 months of age, the mean antibody levels of all calves decreased; a feature they attributed to a low BPI3V antibody response combined with a high rate of maternally-derived BPI3V antibody catabolisation. It is possible that the decrease in peri-weaning BPI3V PP in the present study, in association with a high pre-weaning maximum respiratory score, may be due to a similar phenomenon.

A high maximum pre-weaning respiratory score was also the only risk factor for *H. somni* S/P increase during the peri-weaning period. The *H. somni* ELISA employed in this study detects antibody against EPS, a major component of the *H. somni* biofilm matrix, which is produced in much greater quantities by pathogenic strains during the disease process compared to commensal strains, helping to differentiate between diseased calves and those with antibodies derived from harbouring commensal organisms [23]. Increases in *H. somni* S/P over the peri-weaning period reflect exposure to pathogenic strains of *H. somni* in the preceding weeks. Pan et al. [23] in a study of calves experimentally exposed to *H. somni*, reported antibody peaks to *H. somni* EPS between 3 and 8 weeks post-exposure. The temporal association between respiratory signs (as reflected by a high maximum respiratory score ≥ 8) during the 42 days prior to weaning and the increases in *H. somni* S/P in the peri-weaning period (a range of 4 to 10 weeks after respiratory signs were recorded) concur with the observations of Pan et al. [23] and support their conclusions that assays measuring the antibody response to EPS can identify cattle with disease due to *H. somni* infection.

Levels of antibodies to some BRD pathogens at the beginning of the peri-weaning period were significantly associated with changes in BHV-4 S/P during that period. Higher levels of BRSV and BVDV antibodies (as reflected by BRSV PP and BVDV S/P respectively) at the beginning of weaning were significantly associated (*p* < 0.026) with increases in BHV-4 S/P during the peri-weaning period. Glass et al. [41] have shown that animal genetics influence both the responses to vaccination and infection in cattle. It is possible that higher levels to BVDV and BRSV at the beginning of weaning may reflect a cohort of genetically determined ‘better responders’ in the study population; larger increases in BHV-4 levels in response to exposure during weaning would also be expected among these calves when compared to the poorer responders. It is notable that of the 30 highest responders to BHV-4 during the peri-weaning period, all were Holstein-Friesian from the same farm with the exception of two Jersey calves from a different farm. It is also feasible that these results reflect exposure of the calves to multiple pathogens with humoral responses to BHV-4 elicited at a slower rate when compared to those triggered by exposure to BRSV and BVDV. Although Thiry et al. [42] reported that the immune response of cattle after BHV-4 infection is characterised by low levels of neutralising antibodies, to the authors’ knowledge, studies examining the rate of serological response to BRD pathogens in calves have not been undertaken.

Haptoglobin, a protein produced primarily by hepatocytes following stimulation by proinflammatory cytokines, is part of the acute-phase response to inflammation [43]. While haptoglobin has been identified as a potentially sensitive indicator of BRD in calves owing to its prolonged response [44], this response can be quite variable [45]. Orro et al. [46] concluded that the haptoglobin response requires more profound tissue damage, which is more likely to occur in bacterial rather than viral infections. Young et al. [47], following the screening of cattle at feedlot entry and at 40 and 65 days later, reported that haptoglobin had a poor predictive ability for clinical respiratory disease. In this present study, haptoglobin was only identified as a significant predictor (*p* = 0.007) of the reduction of BHV-1 S/N ratios during the peri-weaning period. Lower S/N ratios reflect higher levels of BHV-1 antibodies, and haptoglobin levels between 1.32 and 1.60 mg/ml were associated with reduced BHV-1 S/N ratios when compared to lower haptoglobin levels (≤1.08 mg/ml). This finding supports a potential role for haptoglobin in identifying exposure of calves to BHV-1.

Data analysis in this study presented some challenges in terms of determining antibody level changes that were significant and in considering the potential effect of MDA. We decided to use all the available information on the change in serological antibody levels (i.e. the change in BRD-specific antibody levels as reflected by the S/P, PP or S/N) rather than assuming that a particular antibody level or antibody level change should be classed as biologically significant. There were a number of reasons for the adoption of this approach. Martin and Bohac [48] reported that such an approach (using ANOVA in their study) was more powerful and more likely to identify significant associations. Windeyer et al. [49] also concluded that the detection of seroconversion was difficult in calves with high levels of MDA as these calves must produce more endogenous antibodies than calves with lower levels of MDA to achieve the four-fold increase in antibodies required for seroconversion. The range of BRD pathogen-specific MDA will vary considerably among calves following the consumption of colostrum [50] but, while BRD pathogen-specific MDA begins to decline from their second month in dairy calves [40], it was estimated by Fulton et al. [51], in a study of non-vaccinated beef calves, that calves may be approximately 6 months of age before MDA against these pathogens is no longer detectable. Calves were enrolled into the present study at a mean age of 19 days and had received colostrum, as reflected by ZST results.

Calf vaccination was necessary in this study, firstly to minimise disease in a group of calves assembled and housed as part of a larger feeding trial and, secondly, as it also reflects the reality of practices on farms where such measures are typically in place. Nevertheless it is difficult to quantify the effect of calf vaccination in this study. MDA inhibits the production of endogenous antibody in response to vaccination [52] and the half-lives of vaccinal antibodies in dairy calves [48] appear to be shorter than those in beef calves [51]. Although calves were vaccinated on arrival, of the BRD pathogen levels examined, only BPI3V and BRSV antibody levels should have been affected by MDA as the BHV-1 ELISA employed does not detect antibodies against the BHV-1 vaccine used. Studies of the serological responses of calves with MDA to inactivated BPI-3 V [53] and inactivated BRSV [54] vaccines have shown that antibody levels continue to decline following initial vaccination and that a second vaccine dose is required to increase levels (BPI3V) or slow the rate of maternal antibody decay (BRSV). The authors thus acknowledge that while MDA and vaccination may have influenced the level of some BRD pathogen-specific antibodies recorded at the beginning of gradual weaning (42 days post-vaccination), they do not consider that these interventions significantly distorted the measured changes in antibody status between the sampling points at the beginning and end of the peri-weaning period. Therefore, this study remains valid in what it reveals about BRD pathogen exposure within housed calves. The authors concede that the control of other aspects of the study (e.g. nutritional status) would suggest that the findings may not be generalisable to the wider population.

## Conclusions

Serological analysis and linear regression modelling has identified associations between a number of intrinsic, management and clinical risk factors such as age at housing, the sharing of pens, pre-weaning respiratory clinical score and serum haptoglobin concentration with exposure to BRD-pathogens among group-housed dairy bull calves. The identification of such factors will contribute to the ongoing evidence-based approach to the prevention of BRD at housing.

## Abbreviations

BCoV: Bovine coronavirus
BHV-1: Bovine herpesvirus 1
BHV-4: Bovine herpesvirus 4
BPI3V: Bovine parainfluenza 3 virus
BRD: Bovine respiratory disease
BRSV: Bovine respiratory syncytial virus
BVDV: Bovine viral diarrhoea virus
EDTA: Ethylenediaminetetraacetic acid
ELISA: Enzyme linked immunosorbent assay
EPS: Exopolysaccharide
Ig: Immunoglobulin
LH: Lithium heparin
PP: Percentage positivity
S/N: Sample to negative value
S/P: Sample to positive ratio
SD: Standard deviation
ZST: Zinc sulphate turbidity
